# A Taxonomic Review of *Clostridium difficile* Phages and Proposal of a Novel Genus, “Phimmp04likevirus”

**DOI:** 10.3390/v7052534

**Published:** 2015-05-21

**Authors:** Katherine R. Hargreaves, Martha R. J. Clokie

**Affiliations:** 1 Department of Infection, Immunity and Inflammation, University of Leicester, Leicestershire LE1 9HN, UK; E-Mail: khargreaves@email.arizona.edu; 2 Department of Ecology and Evolutionary Biology, University of Arizona, AZ 85721, USA

**Keywords:** bacteriophage, *Clostridium difficile*, taxonomy, protein clustering, *Siphoviridae*, *Myoviridae*, small myovirus, bacteriophage phylogeny

## Abstract

Currently, only three phages that infect the medically important bacterium *Clostridium difficile* have been discussed by the International Committee of Viral Taxonomy (ICTV). They are all myoviruses, and have been assigned to the genus “phicd119likevirus”. An additional nine phages have since been described in the literature with their genome data available. The Phicd119likevirus is named after the type species: the myovirus ΦCD119 which was the first *C. difficile* phage to be sequenced. The two additional myoviruses, ϕCD27 and φC2, also fall into this genus based on the similarity of their genome and morphological characteristics. The other nine phages have not been assigned to this genus, and four of them do not fit the criteria for the current taxonomic grouping. We have applied protein clustering analysis to determine their phylogenetic relationships. From these results we propose an additional *myoviridae* genus, that we term “phiMMP04likevirus”.

## 1. Introduction

The sequenced *Clostridium difficile* phages that have been described in the literature are all members of the *Caudovirales* order (tailed phages) and belong to either members of the *Myoviridae* or *Siphoviridae* families [1,2,3,4,5,6,7,8]. The myoviruses can be classified into three distinct morphological groupings based on the capsid diameter and tail lengths [9]: medium sized with capsid diameters between ~60–70 nm and tail lengths of 110–130 nm, long tailed with capsid diameter between ~60 to ~70 nm and tail lengths between 150–260 nm, and small myoviruses with capsid diameters of 40–60 nm and tail lengths of 105–110 nm (Table 1). The phages are all temperate as defined by the presence of predicted integrases, and their genome sizes range from ~31 to ~57 kbps in length with GC% from 28.4% to 30.8% (see Table S1 for genome accession numbers).

**Table 1:** Morphological types of sequenced phages.

Morphotype	Sequenced phages of this morphology	Capsid diameter (nm)	Tail length (nm)	Genome size (kbp)
MM	ΦCD119, φC2, phiCDHM1, phiCDHM19,	60–70	110–130	53–57
SMV	ΦMMP04, phiCDHM11, phiCDHM13, phiCDHM14	40–58	106	~31
LTM	ϕCD27, ΦMMP02	60–70	150–260	~51

Previously, the criteria for inclusion to the myovirus genus phicd119likevirus is the presence of a cytosine-C5 specific DNA methylase (ΦCD119 protein YP_529611.1), and a three gene cassette containing a DnaD (ΦCD119 protein YP_529603.1), a hypothetical protein which is unannotated in the record of ΦCD119 and a single-stranded DNA binding protein (ΦCD119 protein YP_529604.1) [10]. Since this publication an additional six phages have been reported that have not been assigned to this genus, or do not meet these criteria [4,5,6,7,8]. Genome comparisons using either whole genome alignments at the nucleotide level or blastn similarity scores support the distinct groupings of these phages [8,9].

*C. difficile* phages have been the subject of interest due to their ability to further our understanding of the biology of this major human pathogen, and because of their possible exploitation as genetic tools, or their application as novel therapeutics. Indeed, the phages φC2, ϕCD27 and φCD38-2 have been found to modulate toxin production [5,11,12], and φC2 has been demonstrated to perform generalised transduction of a transposon containing antibiotic resistance genes [13]. Phages infecting *C. difficile*, and their products, have been studied for the purposes of developing novel treatments for *C. difficile* infections, this includes the sequenced ϕCD27 and its endolysin [12,14]. In order to make sense of both the aspects of fundamental biology that *C. difficile* phages impact, and to assist in their selection and development as therapeutics, an update on their taxonomic classification is needed.

In order to determine suitable new genera and the defining characteristics we have referenced existing genome comparisons, and have applied protein clustering analysis to twelve *C. difficile* phages.

## 2. Results

### Taxonomic Clusters and Protein Cluster Analysis

**Figure 1:**
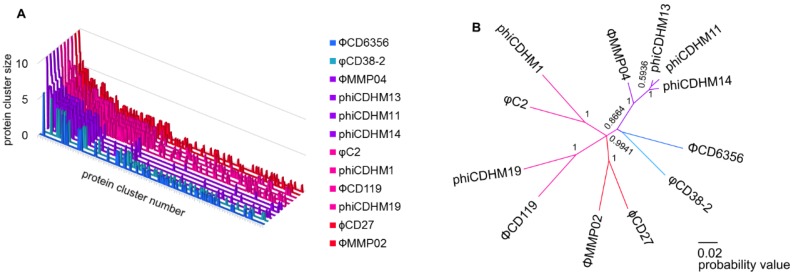
Network of *C. difficile* phages based on conserved protein clusters. (**A**) Conserved protein clusters shown per phage, colour indicates grouping by morphology (blue for siphoviruses, purple for small myovirses, magenta for medium myoviruses and red for long tailed myoviruses). The y axis is cluster size (number of phages represented in each cluster), x axis is protein cluster, sorted by cluster size and z axis is each phage; (**B**) Tree generated using Bayesian phylogenetic inference with node probability based on standard data produced from protein clustering. Taxa clusters correspond to the proposed phage genus groups: phiCD119viruslike with ΦCD119, phiCDHM19, φC2, phiCDHM1, ϕCD27 and ΦMMP02, phiMMP04likevirus with ΦMMP04, phiCDHM13, phiCDHM14 and phiCDHM11. Branch colours correspond to phage particle morphology as in A.

We performed a protein clustering based analysis to determine the core proteomes from the annotated Genbank files for the *C. difficile* phages (Figure 1). A total of 798 protein sequences from the twelve phage genomes (Supplementary Table S1 for details) were compared and 445 protein clusters were identified. The largest cluster included ten of the phages, which were all myoviruses (Figure 1A). This one shared protein cluster is based on a gene encoding an unknown hypothetical protein (reference ORF 3 in ΦCD119). A binary matrix derived from the protein cluster data was used to perform phylogenetic analysis using Bayesian inference in Mr. Bayes (Figure 1B and Supplementary Table S2).

This analysis supports the proposed novel genus and additional members to the existing phicd119ikevirus group. There is a clade for the small myoviruses which is distinct from the medium and long tailed myoviruses with a branch probability of 0.8664 (Figure 1A).

**Figure 2:**
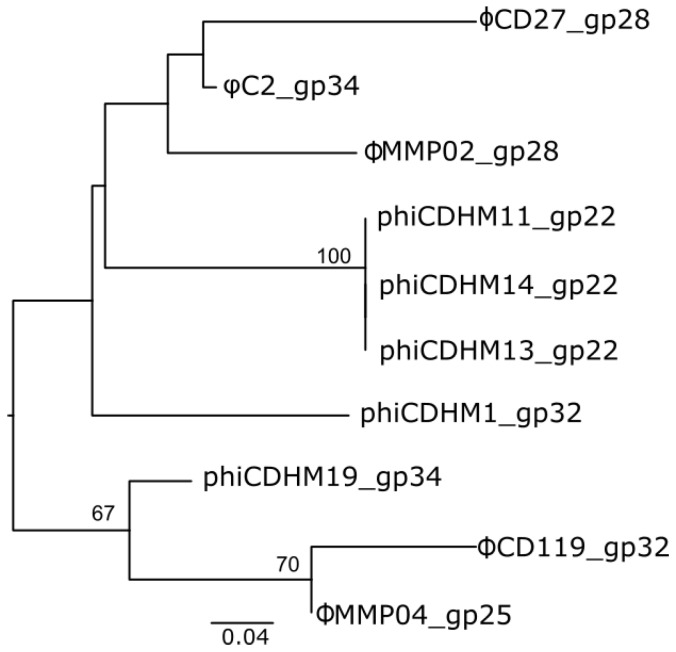
Phylogeny of shared *C. difficile* myovirus protein in cluster 396.

From the clustering analysis it is clear that that there is one protein cluster (PC_376, Table S3) which is common to all the myoviruses examined. This encodes a hypothetical protein 60 aa in length, located within the structural region of the phage genomes. A blastp search using the sequence of CDHM1_gp32 showed a high degree of conservation across multiple *C. difficile* strains, and has usually been annotated as a phage protein. It does share similarity (with an E value of 3e-24 and 85% identity) in a 60 aa region of a 771 aa protein in CD630 (CD630_29521, accession CAJ69777.3) which encodes a predicted ABC transport ATP binding protein. The region sharing similarity is not across the ATP binding predicted domains, or signature motifs, but it suggests that the phage protein could interact with the *C. difficile* cell membrane. This prediction is supported by its location in the phage genome, which is adjacent to other CDSs that are predicted as tail fibers and base plate proteins. Due to its conserved nature across the phage and prophage genomes, this protein may be a specific marker of phage access to *C. difficile*. Phylogenetic analysis using Maximum Likelihood (ML) reveals there is no strong signature across the phage morphotypes (Figure 2).

The nucleotide sequences of shared protein from the ten myoviruses were aligned using MUSCLE and phylogenetic analysis was performed using Maximum likelihood analysis with a Hasegawa-Kishino-Yano [15] nucleotide substitution model and a Gamma distribution rate. The distance between the sequences ranged from 0 to 0.223 and the scale bar indicates number of substitutions per site. The total length of the alignment was 183 positions. The taxa are generally not well supported by bootstrap values, with three clusters having greater than 50; one contains the sequences from the three highly related small myoviruses phiCDHM11, phiCDHM14 and phiCDHM13; the second contains those from ΦCD119 and ΦMMP04 and third expands this cluster to contain phiCDHM19.

## 3. Discussion 

### 3.1. PhiCD119likevirus Clade

The grouping of ΦCD119, φC2, ϕCD27, ΦMMP04, phiCDHM1 and phiCDHM19 into the phicd119likevirus genus is supported by their clustering with a branch probability of 0.9941. These phages are all represented in 1 protein cluster (PC_166, Table S4) which also encodes a protein of unknown function in the DNA replication region of the genomes. There are distinct subgroupings within this genus, with clades containing φC2 and phiCDHM1, which have 19 protein clusters in common (Table S5). These contain mostly proteins that are predicted to be part of the virion particle and an anti-repressor protein (ORF 55 in φC2). The clade containing ΦCD119 and phiCDHM19 also has 19 protein clusters in common (Table S6) which contain a mixture of structural proteins as well as those located in the DNA replication region of the genomes. The two long tailed myoviruses, ϕCD27 and ΦMMP02 cluster together and share 16 protein clusters (Table S7), also mainly comprising of proteins in the structural region with one located in the putative lysogenic conversion module (ORF 41 in ϕCD27).

### 3.2. PhiMMP04likevirus Clade

We propose a new genus, phiMMP04likevirus, which is named after the first published small myovirus, ΦMMP04 [16] and which now includes the three small myoviruses phiCDHM14, phiCDHM11 and phiCDHM13 [8]. This taxonomic grouping is supported by the clustering of the four small myoviruses together, supported by a branch probability of 1. These phages share 16 protein clusters in common (Table S8). The majority of these clusters contain sequences of predicted structural genes, but also three ORFS located in the DNA replication region of the genomes all encoding proteins of unknown functions.

### 3.3. Characteristic Genome Features of the New Genus 

Previously, a gene cassette and putative DNA methylase gene was identified in all the genomes of the three current members of phicd119likevirus [17]. This DNA replication cassette is also present in phiCDHM1, phiCDHM19 and ΦMMP02 and supports the addition of these phages to this genus. However, the genomes of the four short tailed myoviruses do not carry this cassette or DNA methylase. The genomes of all four small myoviruses show a high degree of similarity to one another, and contain a putative ParA homolog. The role of this protein in these phages is unknown: it is located on the antisense strand, downstream of the lysis gene cassette which suggests it may form part of the lysogenic conversion module. ParA has a chromosome partitioning role in bacteria replication [17]. The phages amino acid sequence contains a CbiA protein domain (PF01656), and has homology to SpoOJ regulator protein of *Clostridium sordellii* (Accession CEP41774.1, E value of 4e−116 and identity of 63%), suggesting that it may alternatively influence bacterial sporulation. The two siphoviruses also carry homologs of the ParA-like gene. The small myoviruses and siphoviruses also have in common that their predicted integrases are located on the sense strand following the DNA replication region in the genomes.

In contrast, the small myoviruses differ considerably to the siphoviruses across their structural regions and instead have similarity to ΦCD119 in this region. However, a defining feature is the presence of a gene encoding a predicted Clp protease (a family of serine peptidases, PfaM PF00574) which is not present in ΦCD119. We propose that the presence of these two genes, the ParA homolog and Clp protease, forms the genetic basis of their grouping into the novel genus phiMMP04likevirus, alongside particle morphology dimensions and a genome size ranging between 30–33 kb.

### 3.4. C. Difficile Phages Outside of the Established and Proposed Genera

The two siphoviruses do not fall within either of these genus groups. Between them, the two siphoviruses share four protein clusters in common (Table S9). These four clusters include the endolysin (ORF 28 in ΦCD6356) and holin (ORF 27 ΦCD6356) sequences as well as two from the DNA replication module, one encoding a protein of unknown functions (ORF 47 and ORF 49). The clustering of the two siphoviruses however is not supported by a branch probability, and it may be suitable in the future to further divide these into separate genera depending on future phage discovery. The two siphoviruses both encode the ParA homolog and the genes represented in the protein clusters encoding a protein of unknown function.

### 3.5. Phage and Prophage Sequences in NCBI 

There are several *C. difficile* phage genomes in the NCBI database and many can be assigned these to these proposed genera based on their overall genome identity using blastn and presence of the characteristic genes described above. For example, phiCD111 (accession LN681535) and phiCD146 (accession LN681536) are both similar to the siphoviruses and contain a ParA homolog and the two hypothetical proteins corresponding to ORF 47 and ORF 49 in ΦCD6356. The phages phi481-1 (accession LN681538) and phiCD506 (accession LN681540) can be placed into the phiMMP04viruslike genus due to their genome size and the presence of both a Clp protease homolog and ParA. Where previously described, the morphological characteristics of these phages correspond to these assignments [6,16]. Other phage genomes and prophage sequences in shotgun genome sequences suggest there may be further diversity across phages infecting this species and it is likely that additional modifications to their taxonomic assignments may be needed in the future following their characterisation.

## 4. Materials and Methods

Phage sequences were accessed via NCBI or ENA and their genomes were visualised using Artemis v15.0.0 [18]. Protein clusters were generated in CD-HIT [19] and phylogenetic analysis was performed in MrBayes v3.2.4 [20]. Protein cluster statistics were generated in Microsoft Office Excel and phylogenetic tree imaged using Figtree v1.4.2 [21] and Inkscape v0.91 [22]. Phylogenetic analysis of the conserved amino acid sequences were performed in MEGA v6.0 [23], using alignment with MUSCLE and best Maximum likelihood (ML) model was determined for each analysis. Newick trees were visualised in Figtree.

Phage genomes were searched using blastn against the NCBI nr/nt databases and individual genes using BLASTp and BLASTn.

## Supplementary Files

Supplementary File 1
