# Supplementary material for: A Taxonomic Review of Clostridium difficile Phages and Proposal of a Novel Genus, “Phimmp04likevirus”
Source: Viruses. 2015 May 21;7(5):2534–41. doi: 10.3390/v7052534 (PMC4452919; doi:10.3390/v7052534)
Supplement: Supplementary File 1 [file viruses-07-02534-s001.pdf]

Supplementary Materials

Table S1. *C. difficile* phages and accession numbers.

Phage	Accession number	ORF sequences in Genbank
φC2	NC_009231.1	85
ΦCD119	NC_007917.1	79
phiCDHM1	HG531805	84
phiCDHM19	LK985322	88
ϕCD27	NC_011398.1	75
ΦMMP02	NC_019421.1	76
phiCDHM14	LK985321	50
phiCDHM11	HG798901	47
phiCDHM13	HG796225	50
ΦMMP04	NC_019422.1	51
φCD38-2	NC_015568.1	55
phiCD6356	NC_015262.1	59

Table S2. Protein Clusters with phage representatives.

	phiC D6356	φCD3 8-2	ΦMM P04	phiCDH M13	phiCDH M11	phiCDH M14	φ C2	phiCDH M1	ΦCD 119	phiCDH M19	ϕCD 27	ΦMM P02
0	1											
1		1										
2											1	
3												1
4									1			
5		1										
6										1		
7							1	1				
8										1		
9		1	1	1								
10								1				
11							1	1				
12		1										
13			1	1	1	1						
14		1										
15											1	
16								1				
17										1		1
18			1	1	1	1						
19	1											
20							1			1	1	
21								1				
22			1	1	1	1				1		
23											1	1
24								1				
25							1	1				

Table S2. *Cont.*

	phiC D6356	φCD3 8-2	ΦMM P04	phiCDH M13	phiCDH M11	phiCDH M14	φ C2	phiCDH M1	ΦCD 119	phiCDH M19	ϕCD 27	ΦMM P02
26											1	1
27		1										
28											1	1
29							1					
30					1	1						
31								1				
32											1	
33									1	1		
34											1	1
35		1										
36							1	1				
37				1	1	1			1			
38			1	1	1	1						
39			1									
40							1	1				
41		1										
42											1	
43							1					
44	1											
45	1											
46											1	
47			1	1	1	1						
48								1	1	1		
49	1										1	1
50	1											
51						1	1	1				1
52		1										
53		1										
54									1	1		
55			1	1	1	1			1	1		
56			1	1	1	1						
57												1
58									1	1		
59									1		1	
60							1	1				
61							1			1		1
62											1	1
63							1	1		1		1
64									1			
65											1	1
66											1	
67									1	1		

Table S2. *Cont.*

	phiC<br>D6356	φCD3<br>8-2	ΦMM<br>P04	phiCDH<br>M13	phiCDH<br>M11	phiCDH<br>M14	φ<br>C2	phiCDH<br>M1	ΦCD<br>119	phiCDH<br>M19	ϕCD<br>27	ΦMM<br>P02
68	1											
69									1	1		
70												1
71							1	1				
72							1	1	1	1		1
73								1				
74	1											
75	1											
76							1					
77											1	
78			1	1	1	1	1	1		1	1	1
79							1				1	
80							1		1		1	1
81		1										
82									1		1	
83											1	1
84	1		1									
85		1		1	1	1						
86									1			
87									1			
88	1	1										
89								1				
90		1										
91								1				
92			1	1	1	1						
93											1	
94								1				
95										1		
96								1			1	1
97											1	
98										1		
99									1	1		
100												1
101			1	1	1	1						
102		1										
103								1	1	1		
104				1	1	1						
105		1										
106		1										
107	1											
108									1			
109					1							

Table S2. *Cont.*

	phiC D6356	φCD3 8-2	ΦMM P04	phiCDH M13	phiCDH M11	phiCDH M14	φ C2	phiCDH M1	ΦCD 119	phiCDH M19	ϕCD 27	ΦMM P02
110							1	1			1	1
111							1	1				
112									1			
113									1			
114	1											
115											1	1
116								1				
117									1			
118	1											
119							1	1				
120		1										
121			1	1	1	1				1		
122	1											
123				1	1	1						
124							1					
125									1	1		
126												1
127									1			
128	1											
129			1	1	1	1			1	1		
130							1	1				
131											1	
132							1		1	1		
133	1											
134							1		1	1	1	1
135		1	1	1		1						
136		1										
137						1						
138									1	1		
139	1											
140									1			
141								1				
142								1				
143		1										
144		1										
145		1										
146												1
147							1	1				
148										1	1	
149							1					
150	1											
151										1		

Table S2. *Cont.*

	phiC D6356	φCD3 8-2	ΦMM P04	phiCDH M13	phiCDH M11	phiCDH M14	φ C2	phiCDH M1	ΦCD 119	phiCDH M19	ϕCD 27	ΦMM P02
152	1											
153							1					
154			1									
155							1					
156							1			1	1	
157							1			1	1	
158							1	1		1	1	1
159												1
160		1										
161	1											
162												1
163									1			
164												1
165												1
166							1	1	1	1		
167							1					
168											1	
169											1	
170											1	
171									1	1		
172		1										
173							1	1				
174									1			
175			1	1		1						
176	1											
177											1	1
178			1	1	1	1						
179											1	
180										1		
181										1		
182									1	1		
183				1	1	1						
184				1	1	1						
185											1	1
186	1											
187												1
188							1					
189	1											
190							1	1				
191							1	1				
192		1										
193			1	1	1	1						

Table S2. *Cont.*

	phiC D6356	φCD3 8-2	ΦMM P04	phiCDH M13	phiCDH M11	phiCDH M14	φ C2	phiCDH M1	ΦCD 119	phiCDH M19	ϕCD 27	ΦMM P02
194									1	1		
195									1			
196			1					1		1		
197		1										
198			1	1	1	1			1	1		
199		1										
200							1	1				
201											1	
202			1	1	1	1						
203								1				
204							1		1	1	1	1
205			1	1	1	1						
206												1
207												1
208							1	1				
209									1	1		
210									1			
211							1	1	1	1	1	
212	1											
213		1										
214		1		1	1	1						
215									1	1		
216												1
217									1			
218									1	1		
219							1					
220								1				1
221	1		1									
222									1			
223								1		1		
224				1								
225							1			1	1	
226											1	1
227							1					
228							1					1
229											1	
230								1	1			
231											1	1
232		1		1	1	1						1
233							1		1		1	
234	1											
235									1			

Table S2. *Cont.*

	phiC D6356	φCD3 8-2	ΦMM P04	phiCDH M13	phiCDH M11	phiCDH M14	φ C2	phiCDH M1	ΦCD 119	phiCDH M19	ϕCD 27	ΦMM P02
236	1											
237									1	1		
238											1	1
239		1										
240	1											
241								1				
242		1										
243							1			1	1	
244								1	1	1		
245								1		1		
246												1
247			1	1	1	1						
248									1	1		
249										1		
250		1										
251		1										
252							1	1				
253							1					
254	1											
255			1	1	1	1						
256								1		1	1	
257											1	1
258			1	1	1	1						
259							1		1	1	1	
260	1											
261								1				
262									1			
263		1										
264		1		1	1	1						
265	1											
266											1	
267			1	1	1	1			1	1		
268									1			
269							1					
270	1											
271								1		1		
272									1			
273										1		1
274							1			1		
275											1	
276							1					

Table S2. *Cont.*

	phiC D6356	φCD3 8-2	ΦMM P04	phiCDH M13	phiCDH M11	phiCDH M14	φ C2	phiCDH M1	ΦCD 119	phiCDH M19	ϕCD 27	ΦMM P02
277				1	1	1						
278		1										
279								1		1		
280									1	1		
281	1						1					
282									1			
283										1	1	1
284	1											
285									1			
286	1		1					1		1	1	1
287	1											
288			1	1		1						
289	1											
290								1	1			
291			1									
292			1		1	1						
293	1											
294		1										
295									1			
296									1			
297												1
298		1		1	1	1						
299	1											
300								1				
301			1									
302			1									
303								1				
304									1			
305												1
306	1											
307	1			1	1							
308						1						
309	1	1										
310	1		1	1	1							
311							1	1				
312	1											
313				1	1	1	1		1		1	1
314	1		1									
315							1	1				
316								1	1			
317												1
318									1			

Table S2. *Cont.*

	phiC D6356	φCD3 8-2	ΦMM P04	phiCDH M13	phiCDH M11	phiCDH M14	φ C2	phiCDH M1	ΦCD 119	phiCDH M19	ϕCD 27	ΦMM P02
319									1			
320								1				
321					1							
322									1	1		
323									1			
324												1
325		1										
326								1				
327									1			
328			1									
329								1				
330							1					
331							1					
332										1		
333				1	1	1	1		1		1	1
334									1			
335												1
336										1		
337							1					
338							1					
339							1					
340											1	
341							1	1		1		1
342				1								
343										1		
344									1			1
345	1											
346								1				
347												1
348							1					
349	1											
350				1		1						
351		1										
352											1	
353					1	1						
354												1
355											1	1
356							1					
357								1		1		
358							1					
359								1		1		
360										1		

Table S2. *Cont.*

	phiC D6356	φCD3 8-2	ΦMM P04	phiCDH M13	phiCDH M11	phiCDH M14	φ C2	phiCDH M1	ΦCD 119	phiCDH M19	ϕCD 27	ΦMM P02
361							1	1		1		1
362										1		
363								1	1	1		
364									1	1		
365							1		1	1		
366												1
367						1						
368							1					
369										1		
370							1	1		1	1	1
371											1	
372										1		
373									1			
374		1										
375								1				
376			1	1	1	1	1	1	1	1	1	1
377	1	1	1									
378		1										
379											1	
380				1	1	1						
381							1					
382												1
383							1					
384	1											
385	1											
386	1	1										
387								1				
388								1	1			
389								1	1	1		1
390							1	1		1	1	1
391			1	1	1	1						
392			1									
393	1	1										
394		1										
395											1	
396											1	
397				1	1							
398							1					
399											1	
400	1											
401		1										
402	1											

Table S2. *Cont.*

	phiC D6356	φCD3 8-2	ΦMM P04	phiCDH M13	phiCDH M11	phiCDH M14	φ C2	phiCDH M1	ΦCD 119	phiCDH M19	ϕCD 27	ΦMM P02
403			1	1	1	1						
404							1					
405	1											
406												1
407	1	1	1									
408			1									
409							1					1
410											1	
411	1			1	1							
412							1					
413								1				1
414								1				
415										1		
416											1	1
417	1									1	1	1
418								1				
419										1		
420							1			1	1	
421												1
422											1	
423		1	1									
424								1				
425								1			1	
426								1				1
427					1							
428										1		
429					1							
430		1										
431			1									
432								1			1	
433								1				
434		1										
435		1	1	1								
436		1										
437						1						
438										1		1
439			1									
440			1									
441												1
442						1						
443					1							
444							1					

Table S3. Protein clusters shared between all myoviruses with reference to ΦCD119.

PC	ORF	Product
376	32	unknown

PC = Protein cluster.

Table S4. Protein clusters shared between all phiCDH119likevirus representatives; ΦCD119, phiCDHM19, φC2, phiCDHM1, ϕCD27 and ΦMMP02.

PC	ORF	Product
166	73	unknown

PC = Protein cluster.

Table S5. Protein clusters shared between φC2 and phiCDHM1 with protein clusters with reference to φC2.

PC	ORF	Product
7	20	tape measure protein
11	22	cell wall hydrolase
25	3	portal protein
36	13	tail sheath protein
40	2	TerL
60	25	unknown
71	7	Mjr capsid
111	21	LysM
119	55	Anti-repressor
130	26	unknown
147	6	scaffold protein
173	14	core tail protein
190	16	unknown
191	12	unknown
200	11	unknown
208	9	unknown
252	10	unknown
311	23	unknown
315	15	unknown

PC = Protein cluster.

Table S6. Protein clusters shared between ΦCD119 and phiCDHM19 with to ΦCD119 ORFs.

PC	ORF	Product
33	4	portal
58	5	head protein
125	6	Unknown
138	8	scaffold protein
69	9	capsid

Table S6. *Cont.*

PC	ORF	Product
248	11	Unknown
209	12	unknown
182	13	unknown
54	14	XkdK-like protein
194	15	XkdM-like protein
171	16	XkdMN-like protein
99	21	unknown
322	22	unknown
215	44	unknown
218	45	RepR
364	48	unknown
237	56	unknown
67	77	unknown
280	25	unknown

PC = Protein cluster.

Table S7. Protein clusters shared between ϕCD27 and ΦMMP02 (LTMs) with reference to ϕCD27.

PC	ORF	Product
23	3	TerL
26	13	Unknown
28	2	TerS
34	5	Unknown
62	4	Head Morphological Protein
65	19	Unknown
83	11	Unknown
115	18	LysM
177	21	Unknown
185	10	Unknown
226	6	Unknown
231	9	Unknown
238	20	Unknown
257	8	Unknown
355	12	Unknown
416	41	Unknown

PC = protein cluster.

Table S8. Protein clusters shared between proposed ΦMMP04likevirus group with reference to ΦMMP04.

PC	ORF	Product
13	14	tape measure protein
18	2	Terminse

Table S8. *Cont.*

PC	ORF	Product
38	5	mjr capsid
47	3	portal
56	10	tail sheath protein
92	22	tail fiber
101	4	clp protease
178	8	unknown
193	39	unknown
202	9	unknown
205	12	XkdN-related protein
247	41	Unknown
255	36	Unknown
258	7	head_tail adaptor
391	13	Unknown
403	40	Unknown

PC = Protein cluster.

Table S9. Protein clusters shared between the two siphoviruses φCD38-2 and phiCD6356 with reference to phiCD6356 ORFs.

PC	ORF	Product
88	28	endolysin
309	27	holin
386	47	unknown
393	49	unknown

PC = Protein cluster.

© 2015 by the authors; licensee MDPI, Basel, Switzerland. This article is an open access article distributed under the terms and conditions of the Creative Commons Attribution license (http://creativecommons.org/licenses/by/4.0/).
